# Fabry disease with early-onset ventricular dilation: A case report

**DOI:** 10.1097/MD.0000000000022326

**Published:** 2020-09-25

**Authors:** Jia Tang, Chao Wu, Jian Cao, Liang Wang

**Affiliations:** a Peking Union Medical College Hospital (PUMCH); b Department of Cardiology, Fuwai Hospital, National Center for Cardiovascular Diseases; c Department of Radiology; d Department of Cardiology, Peking Union Medical College Hospital (PUMCH), Chinese Academy of Medical Science (CAMS) and Peking Union Medical College (PUMC), Beijing, China.

**Keywords:** Fabry disease, ventricular dilation, T1 mapping, myocardial fibrosis

## Abstract

**Rationale:**

The most common cardiac involvement of Fabry disease (FD) is left ventricular hypertrophy (LVH), which usually occurs in male patients over the age of 30. In rare cases, it can progress to ventricular dilation in the late stage of the disease.

**Patient concerns:**

A 16-year-old boy presenting with recurrent extremity pain and chest distress was admitted to our hospital. Imaging examinations revealed ventricular dilation.

**Diagnosis:**

α-Galactosidase A enzyme assay and *GLA* gene sequencing confirmed the diagnosis of FD and revealed a novel mutation c.76_77insT.

**Interventions:**

The patient was treated using metoprolol (23.75 mg qd) and angiotensin-converting enzyme inhibitor (fosinopril sodium 5 mg qd). He refused enzyme replacement therapy for financial reasons.

**Outcomes:**

The echocardiography, electrocardiography, renal function, and routine blood and urine tests performed 20 months after the patients discharge from hospital showed no significant changes. The patient reported a slow and gradual decrease in the frequency and degree of pain and chest distress, starting approximately 24 months after discharge.

**Lessons:**

Cardiac involvement of FD can progress rapidly in some cases. Screening for FD should be considered in patients with unexplained ventricular dilation, especially in those with a history of typical FD manifestations.

## 1 Introduction

Fabry disease (FD) is an X-linked recessive lysosomal storage disease caused by a deficiency of the α-galactosidase A enzyme (encoded by the *GLA* gene). The disease can affect multiple organs, including the heart, brain, and kidneys.^[1]^ The most common cardiac involvement is concentric left ventricular hypertrophy (LVH), which usually occurs in male patients over the age of 30.^[2]^ In rare cases, hypertrophic cardiomyopathy can progress to a dilated phase in the late stage of the disease.^[3,4]^ In the present report, we describe the case of a 16-year-old boy diagnosed with FD who developed ventricular dilation. To our knowledge, this is the first reported case of early-onset ventricular dilation in FD, suggesting that cardiac involvement can progress relatively rapidly in some cases.

## 2 Case presentation

A 16-year-old male Chinese patient was admitted to our hospital due to a 4-year history of recurrent upper and lower extremity pain with low-grade fever up to 37.5°C, as well as a 2-year history of chest distress and dyspnea after activity. He complained of an aggravation of chest distress and dyspnea 3 months before admission. No other medical history was reported. Physical examination revealed sporadic angiokeratomas on his waist and back (Fig. 1 C). His blood pressure was normal when he had no extremity pain and could rise to approximately 160/110 mm Hg, with a heart rate of 110 bpm, when the pain attacked. Twelve-lead electrocardiogram showed QT prolongation (QTc = 501 ms) and intraventricular conduction block (QRS = 152 ms) (Fig. 1 A and B). Transthoracic echocardiography (TTE) revealed dilated left and right ventricles, mild mitral and tricuspid valve insufficiency, and an ejection fraction of 61%, with no evidence of myocardial hypertrophy (Table 1, Fig. 1 D). Cardiovascular magnetic resonance (CMR) confirmed that the ventricles were dilated (Fig. 1 F and G, Table 2). No significant abnormalities were found in first-pass perfusion or late gadolinium enhancement (LGE) CMR. Native T1 mapping of the left ventricle is shown in Fig. 1 E. The mean T1 was 1262 ms. Routine blood, urine, and stool tests were all unremarkable, as were hepatic and renal function, erythrocyte sedimentation rate (ESR), C-reactive protein (CRP), joint X-ray and magnetic resonance imaging (MRI), 24-hour urine catecholamine assay, ^99m^Tc-octreotide scan, and ophthalmologic examination. The patient was negative for human leukocyte antigen-B27 (HLA-B27). Leukocyte α-galactosidase A activity was 0.1 nmol/hour/mg protein, which is below the normal range of 29.0 to 64.4 nmol/hour/mg protein. Polymerase chain reaction amplification and Sanger sequencing of the *GLA* exons from genomic DNA of the patient revealed an insertion of 1 nucleotide between positions 76 and 77 in the cDNA (c.76_77insT; Fig. 1 H). The present report is the first to describe this novel mutation, which can generate a premature termination codon at amino acid position 30. The patients mother was heterozygous for the mutation, as shown in Figure 1 I.

**Figure 1:**
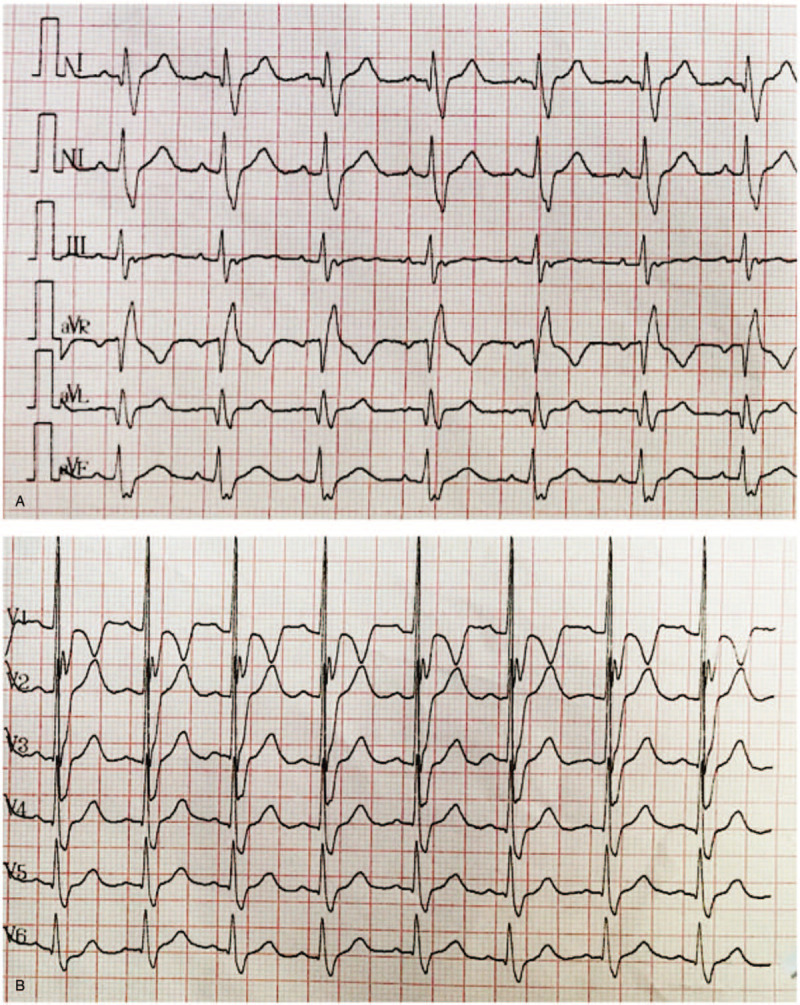
(A) I, II, III, aVR, aVL, aVF leads. (B) V1-V6 leads. (C) Sporadic angiokeratomas on the waist and back. (D) Transthoracic echocardiogram showing left ventricular dilation. LVIDs = left ventricular internal diameter in systole, LVIDd = left ventricular internal diameter in diastole, EDV = end-diastolic volume, ESV = end-systolic volume, FS = short-axis fractional shortening, EF = ejection fraction. (E) Native T1 mapping of the left ventricle. (F) Long-axis four-chamber CMR in diastole. (G) Short-axis two-chamber CMR in diastole. (H) Part of the electropherogram of exon 1 of the *GLA* gene in the patient. The mutation is indicated by the arrow. (I) The same part of the gene in the patients mother, in which the mutation is indicated by the arrow.

**Figure 1 (Continued):**
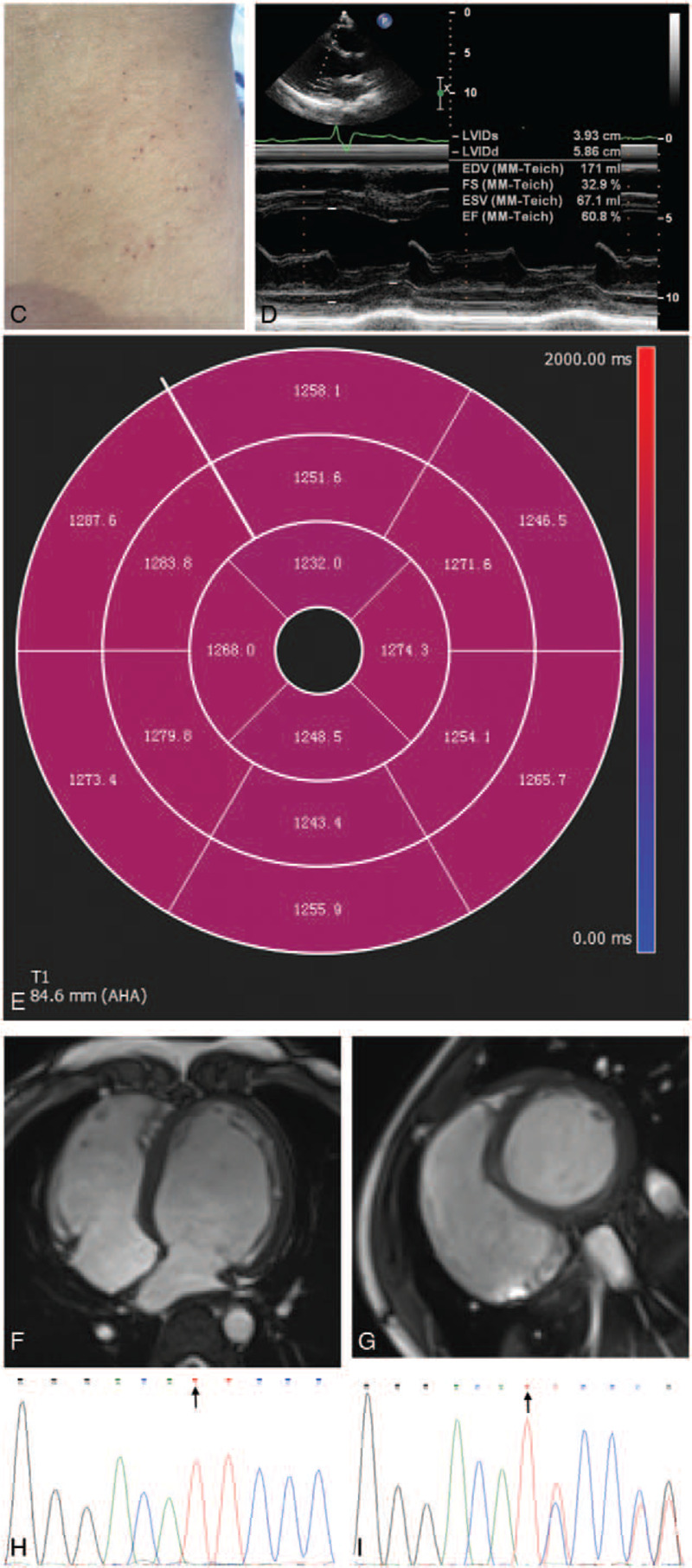
(A) I, II, III, aVR, aVL, aVF leads. (B) V1-V6 leads. (C) Sporadic angiokeratomas on the waist and back. (D) Transthoracic echocardiogram showing left ventricular dilation. LVIDs = left ventricular internal diameter in systole, LVIDd = left ventricular internal diameter in diastole, EDV = end-diastolic volume, ESV = end-systolic volume, FS = short-axis fractional shortening, EF = ejection fraction. (E) Native T1 mapping of the left ventricle. (F) Long-axis four-chamber CMR in diastole. (G) Short-axis two-chamber CMR in diastole. (H) Part of the electropherogram of exon 1 of the *GLA* gene in the patient. The mutation is indicated by the arrow. (I) The same part of the gene in the patients mother, in which the mutation is indicated by the arrow.

**Table 1:**
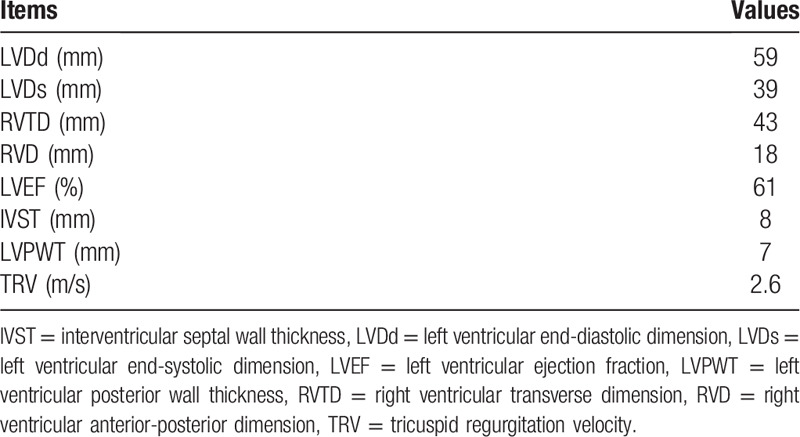
Transthoracic echocardiogram results.

**Table 2:**
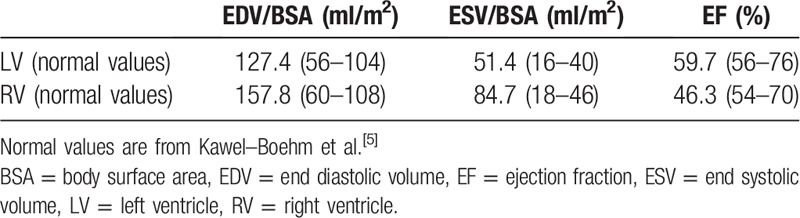
Cardiovascular magnetic resonance results.

The patient was treated using a non-steroidal anti-inflammatory drug (loxoprofen sodium 60 mg q8 hour) after admission, but his extremity pain did not improve. After the definitive diagnosis of FD, his medication was changed to tramadol (100 mg prn), metoprolol (23.75 mg qd), and angiotensin-converting enzyme inhibitor (fosinopril sodium 5 mg qd). Metoprolol and fosinopril sodium were used to delay ventricular remodeling and the possible future decline of renal function. The patient refused enzyme replacement therapy for financial reasons. The tramadol treatment only partially relieved the patients extremity pain, and was discontinued 2 weeks later. The patient was followed up for 29 months. Twenty months after his discharge from the hospital, echocardiography, electrocardiography, renal function, and routine blood and urine tests showed no significant changes. Starting approximately 24 months after his discharge, the patient reported a slow and gradual decrease in the frequency and degree of extremity pain and chest distress, which corroborated previous findings that pain tends to diminish with age.^[6]^

## 3 Discussion

α-Galactosidase A enzyme deficiency in FD results in an abnormal accumulation of glycosphingolipids in various organs. The typical clinical presentations of FD begin in childhood or adolescence and include acroparesthesia, angiokeratomas, gastrointestinal symptoms, corneal opacities, and renal manifestations.^[6]^ Cardiac and cerebrovascular involvement tends to occur in adulthood. The most common cardiac involvement is concentric LVH. Others include myocardial fibrosis, heart failure, coronary artery disease, aortic and mitral valve abnormalities, and conduction abnormalities.^[2,7]^

The present patient was a 16-year-old boy who presented with acroparesthesia, angiokeratomas, and chest distress. The results of the enzyme assay and *GLA* gene sequencing confirmed the diagnosis of FD. Although we identified the same mutation in his mother, there was no family history of FD or related symptoms. Since the most common cardiac manifestation of FD is adult-onset LVH, the results of TTE and CMR showing ventricular dilation confused us before we performed the enzyme assay and gene sequencing. We considered spondyloarthropathy, but excluded this possibility when the ESR, CRP, HLA-B27, and joint X-ray and MRI were negative. Similarly, we excluded pheochromocytoma because the 24-hour urine catecholamine assay and ^99m^Tc-octreotide scan were normal. Although some studies have reported that LVH can progress to a dilated phase in the late stage of the disease,^[3,4]^ the present study constitutes the first reported case of early-onset ventricular dilation in FD. Moreover, we identified a novel mutation: c.76_77insT.

The mechanism underlying the formation of dilated ventricles is uncertain. Since the diagnosis was clear in the present case, we did not perform an endomyocardial biopsy for ethical reasons. However, electrocardiography revealed conduction abnormalities, and TTE showed mitral and tricuspid valve regurgitation, indicating that the ventricular dilation was caused by FD. Conduction abnormalities are thought to result from glycosphingolipid deposition in the conduction system of the heart.^[8]^ Valve regurgitation is also common in the disease.

Another noteworthy finding was the result of CMR T1 mapping. Sado et al reported that the mean T1 in FD patients was approximately 200 ms lower than that in healthy controls.^[9]^ However, in the present case, the mean T1 was only slightly lower than the normal value, which is 1300 ms in our hospital. Sado et al suggested that the T1 lowering is caused by glycosphingolipid deposition and water-lipid interaction. However, they also found that, in some segments with myocardial fibrosis, the local T1 could be normal or even raised because of regional fibrosis. Moreover, several studies have shown that, regardless of the LGE result, the mean T1 in patients with dilated cardiomyopathy is higher than that in normal controls, suggesting diffuse fibrosis.^[10,11]^ Therefore, it may be that the mean T1 in the present patient was slightly reduced because his lesions had progressed to a myocardial scarring phase, in which diffuse fibrosis can elevate or pseudonormalize T1. Diffuse fibrosis may also explain the normal LGE-CMR result.

The natural course of FD cardiac variant progresses from glycosphingolipid deposition to myocardial fibrosis, with thinning of the affected walls.^[12]^ In the present case report, we further hypothesized that diffuse fibrosis itself causes ventricle dilation. It follows that, depending on the speed of progression of fibrosis, the dilated phase may occur in the early stages of the disease, not only in the late stages as other cases have suggested. Therefore, in patients with ventricular dilation of unknown causes, comprehensive history taking and thorough physical examination should be performed. If typical manifestations of FD exist, such as acroparesthesia, angiokeratomas, or renal involvement, then a diagnosis of FD should be considered, and an α-galactosidase A enzyme assay should be conducted to verify the diagnosis.

## 4 Informed consent

Written informed consent was obtained from the patient and his parents for publication of this case report and any accompanying images. The study was approved by the local ethics committee of Peking Union Medical College.

## Author contributions

**Resources:** Liang Wang.

**Supervision:** Liang Wang.

**Visualization:** Jian Cao.

**Writing – original draft:** Jia Tang, Chao Wu.

**Writing – review & editing:** Liang Wang.

## References

[1] Germain DP Fabry disease. Orphanet J Rare Dis 2010;5:30. 21092187 10.1186/1750-1172-5-30 PMC3009617

[2] Kampmann C Linhart A Baehner F Onset and progression of the Anderson-Fabry disease related cardiomyopathy. Int J Cardiol 2008;130:367–73. 18572264 10.1016/j.ijcard.2008.03.007

[3] Fukuzawa K Yoshida A Onishi T Dilated phase of hypertrophic cardiomyopathy caused by Fabry disease with atrial flutter and ventricular tachycardia. J Cardiol 2009;54:139–43. 19632534 10.1016/j.jjcc.2008.10.004

[4] Igawa O Miake J Hisatome I Ventricular tachycardias and dilated cardiomyopathy caused by Fabry disease. Pacing Clin Electrophysiol 2005;28:1142–3. 16221278 10.1111/j.1540-8159.2005.00241.x

[5] Kawel-Boehm N Maceira A Valsangiacomo-Buechel ER Normal values for cardiovascular magnetic resonance in adults and children. J Cardiovasc Magn Reson 2015;17:29. 25928314 10.1186/s12968-015-0111-7 PMC4403942

[6] MacDermot KD Holmes A Miners AH Anderson-Fabry disease: clinical manifestations and impact of disease in a cohort of 98 hemizygous males. J Med Genet 2001;38:750–60. 11694547 10.1136/jmg.38.11.750 PMC1734761

[7] Patel MR Cecchi F Cizmarik M Cardiovascular events in patients with Fabry disease: natural history data from the Fabry Registry. J Am Coll Cardiol 2011;57:1093–9. 21349401 10.1016/j.jacc.2010.11.018

[8] Ikari Y Kuwako K Yamaguchi T Fabry's disease with complete atrioventricular block: histological evidence of involvement of the conduction system. Br Heart J 1992;68:323. 1389767 10.1136/hrt.68.9.323 PMC1025080

[9] Sado DM White SK Piechnik SK Identification and assessment of Anderson-Fabry disease by cardiovascular magnetic resonance noncontrast myocardial T1 mapping. Circ Cardiovasc Imaging 2013;6:392–8. 23564562 10.1161/CIRCIMAGING.112.000070

[10] Dass S Suttie Joseph J Piechnik Stefan K Myocardial tissue characterization using magnetic resonance noncontrast T1 mapping in hypertrophic and dilated cardiomyopathy. Circ Cardiovasc Imaging 2012;5:726–33. 23071146 10.1161/CIRCIMAGING.112.976738

[11] Puntmann VO Voigt T Chen Z Native T1 mapping in differentiation of normal myocardium from diffuse disease in hypertrophic and dilated cardiomyopathy. JACC Cardiovas Imaging 2013;6:475–84. 10.1016/j.jcmg.2012.08.019 23498674

[12] Seydelmann N Wanner C Störk S Fabry disease and the heart. Best Pract Res Clin Endocrinol Metab 2015;29:195–204. 25987173 10.1016/j.beem.2014.10.003

